# Supplementary material for: Safety assessment of sodium zirconium cyclosilicate: A FAERS-based disproportionality analysis
Source: PLoS One. 2025 Mar 25;20(3):e0320585. doi: 10.1371/journal.pone.0320585 (PMC11936284; doi:10.1371/journal.pone.0320585)
Supplement: S1 Table — (DOCX) [file pone.0320585.s001.docx]

**S1 Table: Contingency table for AE signal detection.**

|  | SZC-related AEs | Non-SZC-related AEs | Total |
| --- | --- | --- | --- |
| SZC | a | b | a + b |
| Non-SZC | c | d | c + d |
| Total | a + c | b + d | N = a + b + c + d |

a, number of reports containing both SZC and target AEs.

b, number of reports containing other AEs of SZC.

c, number of reports containing the target AEs of other drugs.

d, number of reports containing other drugs and other AEs.

Abbreviations: SZC, sodium zirconium cyclosilicate; AEs, adverse events.
