# Supplementary material for: Safety assessment of sodium zirconium cyclosilicate: A FAERS-based disproportionality analysis
Source: PLoS One. 2025 Mar 25;20(3):e0320585. doi: 10.1371/journal.pone.0320585 (PMC11936284; doi:10.1371/journal.pone.0320585)
Supplement: S2 Table — (DOCX) [file pone.0320585.s002.docx]

**S2 Table: Four methods, formulas and thresholds.**

| Method | Formula | ﻿Threshold |
| --- | --- | --- |
| ROR | $ROR=\frac{\mathrm{ad}}{\mathrm{bc}}$ | N≥ 3  lower limit of 95%CI > 1 |
|  | $95\%CI=e^{ln(ROR)\pm1.96\sqrt{\frac{1}{a}+\frac{1}{b}+\frac{1}{c}+\frac{1}{d}}}$ |  |
| PRR | $PRR=\frac{a / (a+b)}{c / (c+d)}$ | N≥ 3  χ^2^≥4  ﻿lower limit of 95%CI > 1 |
|  | $95\%CI=e^{ln(PRR)\pm1.96\sqrt{\frac{1}{a}+\frac{1}{b}+\frac{1}{c}+\frac{1}{d}}}$ |  |
|  | $\chi^{2}=\frac{(a+b+c+d){(ad-bc)}^{2}}{(a+b)(c+d)(a+c)(b+d)}$ |  |
| BCPNN | $IC=\log_{2} \frac{a(a+b+c+d)}{(a+b)(a+c)}$ | IC025>0 |
|  | $95\%CI=E(IC)\pm2\times\sqrt{V(IC)}$ |  |
| EBGM | $EBGM=\frac{a(a+b+c+d)}{\left( a+c \right)(a+b)}$ | EBGM05>2 |
|  | $95\%CI=e^{ln(EBGM)\pm1.96\sqrt{\frac{1}{a}+\frac{1}{b}+\frac{1}{c}+\frac{1}{d}}}$ |  |

Abbreviations: ROR, reporting odds ratio; PRR, proportional reporting ratio; BCPNN, bayesian confidence propagation neural network; EBGM, empirical Bayesian geometric mean; CI, confidence interval; χ2,chi-squared; IC, information component; IC025, the lower bound of 95% CI; EBGM05, the lower bound of 95% CI; N, the number of reports.
