# Supplementary material for: Safety assessment of sodium zirconium cyclosilicate: A FAERS-based disproportionality analysis
Source: PLoS One. 2025 Mar 25;20(3):e0320585. doi: 10.1371/journal.pone.0320585 (PMC11936284; doi:10.1371/journal.pone.0320585)
Supplement: S3 Table — (DOCX) [file pone.0320585.s003.docx]

**S3 Table: Signal strength of reports of SZC at the SOC level in the FAERS database.**

| **System organ class (SOC)** | **Case number** | **ROR(95% CI)** | **PRR(95% CI)** | χ**^2^** | **IC(IC025)** | **EBGM(EBGM05)** |
| --- | --- | --- | --- | --- | --- | --- |
| General disorders and administration site conditions | 620 | **1.97(1.79, 2.16)** | **1.67(1.57, 1.77)** | **205.3** | **0.74(0.61)** | 1.67(1.55) |
| Gastrointestinal disorders | 324 | **2.11(1.87, 2.38)** | **1.93(1.75, 2.13)** | **159.11** | **0.95(0.78)** | 1.93(1.75) |
| Investigations | 250 | **2.19(1.92, 2.50)** | **2.04(1.81, 2.29)** | **141.74** | **1.03(0.84)** | 2.04(1.83) |
| Injury, poisoning and procedural complications | 143 | 0.55(0.47, 0.65) | 0.58(0.5, 0.68) | **48.37** | -0.78(-1.02) | 0.58(0.51) |
| Metabolism and nutrition disorders | 115 | **2.91(2.41, 3.51)** | **2.8(2.35, 3.34)** | **135.75** | **1.48(1.22)** | **2.8(2.39)** |
| Cardiac disorders | 107 | **2.61(2.15, 3.17)** | **2.53(2.12, 3.02)** | **100.66** | **1.34(1.06)** | **2.52(2.15)** |
| Nervous system disorders | 82 | 0.50(0.40, 0.62) | 0.52(0.42, 0.65) | **39.12** | -0.94(-1.26) | 0.52(0.43) |
| Renal and urinary disorders | 73 | **1.70(1.34, 2.15)** | **1.67(1.35, 2.07)** | **20.19** | **0.74(0.41)** | 1.67(1.38) |
| Infections and infestations | 57 | 0.48(0.37, 0.62) | 0.49(0.38, 0.63) | **32.12** | -1.03(-1.41) | 0.49(0.39) |
| Skin and subcutaneous tissue disorders | 53 | 0.41(0.31, 0.54) | 0.43(0.33, 0.57) | **43.71** | -1.23(-1.62) | 0.43(0.34) |
| Respiratory, thoracic and mediastinal disorders | 50 | 0.51(0.39, 0.68) | 0.52(0.4, 0.68) | **22.98** | -0.94(-1.34) | 0.52(0.41) |
| Musculoskeletal and connective tissue disorders | 44 | 0.40(0.29, 0.53) | 0.41(0.31, 0.55) | **39.7** | -1.29(-1.72) | 0.41(0.32) |
| Vascular disorders | 28 | 0.70(0.48, 1.02) | 0.7(0.48, 1.02) | 3.55 | -0.51(-1.04) | 0.7(0.52) |
| Psychiatric disorders | 24 | 0.20(0.13, 0.30) | 0.21(0.14, 0.31) | **75.44** | -2.25(-2.82) | 0.21(0.15) |
| Blood and lymphatic system disorders | 11 | 0.30(0.17, 0.55) | 0.31(0.17, 0.56) | **17.44** | -1.7(-2.52) | 0.31(0.19) |
| Neoplasms benign, malignant and unspecified | 11 | 0.14(0.08, 0.25) | 0.14(0.08, 0.25) | **59.82** | -2.83(-3.64) | 0.14(0.09) |
| Eye disorders | 8 | 0.20(0.10, 0.39) | 0.2(0.1, 0.4) | **26.31** | -2.33(-3.27) | 0.2(0.11) |
| Hepatobiliary disorders | 8 | 0.45(0.23, 0.91) | 0.46(0.23, 0.91) | **5.23** | -1.13(-2.08) | 0.46(0.26) |
| Reproductive system and breast disorders | 6 | 0.45(0.20, 1.00) | 0.45(0.2, 1.01) | **4.03** | -1.15(-2.22) | 0.45(0.23) |
| Immune system disorders | 6 | 0.23(0.10, 0.51) | 0.23(0.1, 0.51) | **15.61** | -2.12(-3.19) | 0.23(0.12) |
| Ear and labyrinth disorders | 6 | 0.68(0.31, 1.52) | 0.69(0.31, 1.54) | 0.87 | -0.55(-1.62) | 0.69(0.35) |

Abbreviations: SOC, system organ class; AEs, adverse events; SZC, sodium zirconium cyclosilicate.
