# Supplementary material for: Safety assessment of sodium zirconium cyclosilicate: A FAERS-based disproportionality analysis
Source: PLoS One. 2025 Mar 25;20(3):e0320585. doi: 10.1371/journal.pone.0320585 (PMC11936284; doi:10.1371/journal.pone.0320585)
Supplement: S4 Table — (DOCX) [file pone.0320585.s004.docx]

**S4 Table: The signal strength of AEs at PT level in male subgroup using ROR, PRR, BCPNN, and EBGM.**

| **Male** | | | | | | |
| --- | --- | --- | --- | --- | --- | --- |
| **Preferred terms** | **Case number** | **ROR(95% CI)** | **PRR(95% CI)** | χ**^2^** | **IC(IC025)** | **EBGM(EBGM05)** |
| Computerised tomogram abnormal | 3 | 148.38(47.28, 465.67) | 147.93(47.46, 461.07) | 429.92 | 7.18(5.75) | 145.28(55.8) |
| Blood potassium abnormal | 6 | 124.09(55.27, 278.59) | 123.34(55.22, 275.49) | 717.13 | 6.92(5.84) | 121.49(61.75) |
| Scrotal oedema | 3 | 104.25(33.32, 326.17) | 103.94(33.35, 323.96) | 301.94 | 6.68(5.25) | 102.62(39.51) |
| Blood potassium increased | 25 | 81.31(54.56, 121.17) | 79.27(53.56, 117.31) | 1913.75 | 6.29(5.73) | 78.5(56.22) |
| Product preparation issue | 3 | 33.73(10.83, 105.01) | 33.63(10.79, 104.82) | 94.59 | 5.07(3.65) | 33.49(12.95) |
| Oedema | 22 | 30.85(20.2, 47.12) | 30.19(20, 45.56) | 618.96 | 4.91(4.31) | 30.08(21.1) |
| Hypokalaemia | 15 | 20.93(12.56, 34.88) | 20.63(12.39, 34.34) | 279.62 | 4.36(3.65) | 20.58(13.42) |
| Ileus | 3 | 15.55(5, 48.35) | 15.51(4.98, 48.34) | 40.64 | 3.95(2.53) | 15.48(5.99) |
| Hyperkalaemia | 12 | 14.82(8.38, 26.2) | 14.65(8.3, 25.86) | 152.52 | 3.87(3.08) | 14.63(9.08) |
| Death | 218 | 13.75(11.83, 15.99) | 10.93(9.72, 12.29) | 2004.5 | 3.45(3.24) | 10.92(9.62) |
| Cardiac failure congestive | 9 | 11.24(5.83, 21.68) | 11.15(5.84, 21.29) | 83.08 | 3.48(2.58) | 11.13(6.43) |
| Metabolic acidosis | 7 | 10.57(5.03, 22.25) | 10.51(4.99, 22.13) | 60.18 | 3.39(2.39) | 10.49(5.63) |
| Cardiac failure | 15 | 8.92(5.35, 14.86) | 8.8(5.29, 14.65) | 103.76 | 3.14(2.42) | 8.79(5.74) |
| Constipation | 26 | 7.96(5.39, 11.75) | 7.77(5.35, 11.28) | 153.83 | 2.96(2.41) | 7.77(5.61) |
| Incorrect route of product administration | 4 | 7.87(2.95, 21.03) | 7.85(2.95, 20.92) | 23.88 | 2.97(1.7) | 7.84(3.45) |
| Oedema peripheral | 10 | 6.76(3.62, 12.61) | 6.7(3.65, 12.3) | 48.54 | 2.74(1.89) | 6.7(3.98) |
| Intentional product misuse | 10 | 6.19(3.32, 11.54) | 6.13(3.34, 11.25) | 43.02 | 2.62(1.76) | 6.13(3.64) |
| Swelling | 6 | 5.21(2.33, 11.63) | 5.18(2.32, 11.57) | 20.27 | 2.37(1.3) | 5.18(2.65) |
| Joint swelling | 7 | 4.64(2.2, 9.76) | 4.61(2.19, 9.71) | 19.82 | 2.2(1.2) | 4.61(2.47) |
| Blood pressure increased | 11 | 4.47(2.47, 8.11) | 4.43(2.46, 7.98) | 29.32 | 2.15(1.33) | 4.43(2.7) |
| Abdominal Distension | 6 | 4.41(1.98, 9.85) | 4.39(1.97, 9.81) | 15.73 | 2.13(1.06) | 4.39(2.24) |
| Weight increased | 11 | 3.68(2.03, 6.67) | 3.65(2.03, 6.57) | 21.24 | 1.87(1.05) | 3.65(2.22) |
| Renal failure | 11 | 3.32(1.83, 6.01) | 3.29(1.83, 5.92) | 17.59 | 1.72(0.9) | 3.29(2) |
