# Supplementary material for: Safety assessment of sodium zirconium cyclosilicate: A FAERS-based disproportionality analysis
Source: PLoS One. 2025 Mar 25;20(3):e0320585. doi: 10.1371/journal.pone.0320585 (PMC11936284; doi:10.1371/journal.pone.0320585)
Supplement: S5 Table — (DOCX) [file pone.0320585.s005.docx]

**S5 Table: The signal strength of AEs at PT level in female subgroup using ROR, PRR, BCPNN, and EBGM.**

| **Female** | | | | | | |
| --- | --- | --- | --- | --- | --- | --- |
| **Preferred terms** | **Case number** | **ROR(95% CI)** | **PRR(95% CI)** | **χ^2^** | **IC(IC025)** | **EBGM(EBGM05)** |
| Blood sodium increased | 4 | 180.76(67.34, 485.23) | 179.66(67.43, 478.7) | 704.25 | 7.48(6.2) | 178.04(77.93) |
| Computerised tomogram abnormal | 3 | 165.69(53.05, 517.54) | 164.94(52.92, 514.08) | 484.79 | 7.35(5.93) | 163.58(63.07) |
| Blood potassium abnormal | 4 | 136.19(50.79, 365.19) | 135.37(50.81, 360.69) | 529.87 | 7.07(5.8) | 134.45(58.9) |
| Blood potassium increased | 12 | 101(56.98, 179.04) | 99.17(56.17, 175.08) | 1160.56 | 6.62(5.83) | 98.68(61.12) |
| Faecaloma | 3 | 59.27(19.03, 184.56) | 59(18.93, 183.89) | 170.56 | 5.88(4.46) | 58.83(22.74) |
| Product residue present | 4 | 39.49(14.76, 105.64) | 39.26(14.73, 104.61) | 148.85 | 5.29(4.02) | 39.18(17.2) |
| Ileus | 3 | 36.22(11.64, 112.71) | 36.06(11.57, 112.39) | 102.09 | 5.17(3.75) | 35.99(13.92) |
| Hypokalaemia | 16 | 34.12(20.77, 56.06) | 33.32(20.41, 54.39) | 501.08 | 5.06(4.36) | 33.26(21.96) |
| Cardiac failure congestive | 10 | 26.26(14.06, 49.06) | 25.87(14.09, 47.5) | 238.95 | 4.69(3.83) | 25.84(15.32) |
| Glomerular filtration rate decreased | 3 | 25.74(8.27, 80.07) | 25.62(8.22, 79.85) | 70.91 | 4.68(3.26) | 25.59(9.9) |
| Oedema | 11 | 22.83(12.58, 41.45) | 22.46(12.48, 40.44) | 225.51 | 4.49(3.66) | 22.44(13.63) |
| Blood potassium decreased | 7 | 19.19(9.11, 40.42) | 18.99(9.02, 39.99) | 119.27 | 4.25(3.24) | 18.98(10.17) |
| Death | 110 | 19.03(15.51, 23.36) | 16.01(13.42, 19.1) | 1563.56 | 4(3.71) | 16(13.48) |
| Hyperkalaemia | 4 | 16.97(6.35, 45.38) | 16.88(6.34, 44.98) | 59.72 | 4.08(2.81) | 16.86(7.41) |
| Ascites | 3 | 12.12(3.9, 37.7) | 12.07(3.87, 37.62) | 30.46 | 3.59(2.17) | 12.06(4.67) |
| Feeding disorder | 3 | 10.66(3.43, 33.14) | 10.61(3.4, 33.07) | 26.12 | 3.41(1.99) | 10.61(4.1) |
| Metabolic acidosis | 3 | 9.5(3.05, 29.53) | 9.46(3.04, 29.48) | 22.7 | 3.24(1.82) | 9.46(3.66) |
| Cardiac failure | 6 | 8.91(3.99, 19.92) | 8.84(3.96, 19.74) | 41.76 | 3.14(2.07) | 8.84(4.51) |
| Renal disorder | 4 | 8.65(3.23, 23.11) | 8.6(3.23, 22.91) | 26.87 | 3.1(1.83) | 8.6(3.78) |
| Constipation | 20 | 8.51(5.45, 13.28) | 8.28(5.38, 12.74) | 128.42 | 3.05(2.42) | 8.28(5.7) |
| Oedema peripheral | 5 | 5.85(2.43, 14.11) | 5.82(2.41, 14.06) | 19.97 | 2.54(1.38) | 5.82(2.78) |
| Flatulence | 3 | 5.39(1.73, 16.77) | 5.37(1.72, 16.74) | 10.68 | 2.43(1.01) | 5.37(2.08) |
| Fluid retention | 3 | 5.34(1.72, 16.6) | 5.32(1.71, 16.58) | 10.53 | 2.41(0.99) | 5.32(2.06) |
| Gastrointestinal haemorrhage | 3 | 5.32(1.71, 16.55) | 5.3(1.7, 16.52) | 10.48 | 2.41(0.99) | 5.3(2.05) |
| Diarrhoea | 24 | 3.18(2.12, 4.78) | 3.1(2.09, 4.59) | 34.54 | 1.63(1.06) | 3.1(2.2) |
