# Supplementary material for: Safety assessment of sodium zirconium cyclosilicate: A FAERS-based disproportionality analysis
Source: PLoS One. 2025 Mar 25;20(3):e0320585. doi: 10.1371/journal.pone.0320585 (PMC11936284; doi:10.1371/journal.pone.0320585)
Supplement: S6 File — (DOCX) [file pone.0320585.s006.docx]

**The READUS-PV checklist for abstracts**

| **Section and topic** | **Item #** | **Checklist item** | **Location where item is reported** |
| --- | --- | --- | --- |
| Background | *1a* | *State the aim/rationale for performing the study.* | *Row17-18* |
|  | *1b* | *Specify the adverse event(s) and/or the drug(s) under study, when applicable.* | *Row16* |
|  | *1c* | *Specify the specific population or setting, when applicable.* | *All ages* |
| Methods | *2a* | *Identify the study as a “disproportionality analysis” and specify the type of data used.* | *Row20* |
|  | *2b* | *Specify the name of the database(s) used and the type of access.* | *Row18-19* |
|  | *2c* | *Specify the timeframe and geographical region, when applicable.* | *Row20* |
|  | *2d* | *Specify the disproportionality measure(s) used and their statistical significance threshold(s).* | *Row21-23* |
|  | *2e* | *Specify if a case-by-case analysis is performed.* | *Row24-25* |
| Results | *3* | *Report main findings including their precision (e.g., 95% confidence intervals), together with a short summary of the case-by-case analysis.* | *Row25-33* |
| Conclusion | *4a* | *Clearly report key conclusions.* | *Row33-34* |
|  | *4b* | *Acknowledge that the disproportionality analysis is a hypothesis generating or refinement approach.* | *Row36-37* |
|  | *4c* | *State the implications and clinical relevance of the findings.* | *Row34-35* |

**The READUS-PV checklist for manuscript**

| **Section and topic** | **Item #** | **Checklist item** | **Location where item is reported** |
| --- | --- | --- | --- |
| **Title** |  |  |  |
|  | *1a* | *If disproportionality analyses are a prominent component of the published study, the study should be identified as a “disproportionality analysis”. The type of data and name of the database(s) should be specified.* | *Row2* |
|  | *1b* | *Report the name of adverse event(s) and/or drug(s) under study, when applicable.* | *Row1* |
| **Introduction** |  |  |  |
| Background | *2a* | *Describe the drug(s) and its utilization, the nature of the adverse event(s) under study and its frequency, and the existing knowledge on the drug-event combination.* | *Row51-55* |
|  | *2b* | *Specify the rationale for performing the analysis, e.g., as part of routine pharmacovigilance, to investigate an overall safety profile, or to assess a pre-specified hypothesis.* | *Row56-58* |
|  | *2c* | *Explain why ICSR databases and disproportionality analysis are suitable to fill the knowledge gap.* | *Row62-66* |
| Objectives | *3* | *State specific objectives, identifying the adverse event(s), the drug(s), and the reference group, including any pre-specified hypothesis, if applicable.* | *Row67-71* |
| **Methods** |  |  |  |
| Study design | *4a* | *Identify the study (i.e., “disproportionality analysis”) and the type of data used (e.g., “individual case safety reports”).* | *Row99, Row81* |
|  | *4b* | *Provide an outline of the entire study design, including primary and sensitivity analyses performed, and other designs such as case-by-case analysis or literature review.* | *Row99-119, Fig 1* |
| Data description, access, and pre-processing | *5a* | *Specify the name of the database(s), the database(s) custodian, and the coverage. Specify the type/number of drugs included within the database and the thesaurus, taxonomies, or ontologies used for coding drugs and events.* | *Row74-80* |
|  | *5b* | *Specify the extraction dates and describe and justify all choices used for data pre-processing, including any data transformation or exclusion, if appropriate.* | *Row81-86* |
| Variables definition | *6a* | *Describe the study population, including any restriction.* | *Row81-86* |
|  | *6b* | *Describe the nature and the meaning of key variables assessed in the work.* | *Row93-96* |
|  | *6c* | *Specify and justify any grouping of drugs or events. For drugs, specify and justify whether active ingredients/trade names/salts were considered and/or the selected role.* | *Row88-93* |
|  | *6d* | *Describe any additional data source used, the type of data, and how they interact with ICSRs.* | *Row89-93* |
| Statistical methods | *7a* | *Present any descriptive analysis performed, specifying variables investigated, statistical tests, and significance thresholds.* | *Row99-105,S1 Table* |
|  | *7b* | *Describe the measure(s) selected for the disproportionality analysis including any threshold used to identify signals of disproportionate reporting. Explain the reason for this choice if applicable.* | *S2 Table* |
|  | *7c* | *Clearly describe any sensitivity analysis and any tool to control confounding, including any restriction, subgroup, stratification, adjustment, or interaction.* | *Row93-108* |
|  | *7d* | *Specify the variables and methods used for the case-by-case analysis, including any algorithm or criteria used to assess causality, if performed.* | *Row99-108* |
|  | *7e* | *Specify any statistical methods used for other data sources.* | *Row120-124* |
| **Results** |  |  |  |
| Participants | *8a* | *Specify the number of individual case safety reports included at each stage, including reasons for exclusion.* | *Row131-133,Table 1* |
|  | *8b* | *Provide key demographic and clinical characteristics of cases, if possible comparing cases with any appropriate reference group.* | *Row134-144* |
| Disproportionality analysis | *9* | *Present all results including confidence intervals. Present also results of sensitivity analyses, if performed.* | *Table 2 and S3 Table* |
| Case-by-case analysis | *10* | *Present the case-by-case analysis of key variables. Present the causality assessment, if applicable.* | *Row188-192* |
| **Discussion** |  |  |  |
| Key results | *11* | *Discuss key results with reference to study objectives and contextualize them within the current literature and other consulted sources. Clearly discriminate between expected reactions and emerging safety signals.* | *Row212-226* |
| External validity | *12a* | *Discuss the external validity of the results to the general population.* | *Row327-346* |
|  | *12b* | *Discuss the potential relevance of results in clinical practice* | *Row370-373* |
|  | *12c* | *Propose further study designs if applicable* | *Row370-373* |
| Limitations | *13* | *Present general limitations, making clear that disproportionality analysis alone cannot prove causation or measure incidence, and specific limitations, including confounding and reporting bias and efforts to mitigate them.* | *Row359-370* |
| **Declarations** |  |  |  |
|  | *14a* | *Provide the source of funding/sponsorship and the role of the funders/sponsors for the present study and for any original study on which the present article is based.* | *Row383-390* |
|  | *14b* | *Clearly identify potential commercial and intellectual conflicts of interest (e.g., link to any drug/event investigated, whether financial, legal action, or software used).* | *Row392-396* |
|  | *14c* | *Declare any institutional approval needed or granted in the investigation.* | *Row96-98* |
|  | *14d* | *Include a statement on data availability, code availability (including the version of the statistical software used), and protocol registration.* | *Row402-404* |
