# Supplementary material for: Safety assessment of sodium zirconium cyclosilicate: A FAERS-based disproportionality analysis
Source: PLoS One. 2025 Mar 25;20(3):e0320585. doi: 10.1371/journal.pone.0320585 (PMC11936284; doi:10.1371/journal.pone.0320585)
Supplement: Attachment — Submitted filename: Response to the academic editor and reviewers.docx [file pone.0320585.s008.docx]

Dear Editor and Reviewers,

Thank you for perspective on our work. Your comments have been extremely helpful for improving the quality of our manuscript. We have reviewed and revised the reference list. Now we ensure that it is complete and correct. Words shown in red are the changes we have made in the manuscript. We have also provided our point-by-point response to the comments from each reviewer, and we have highlighted the changes in the revised manuscript. The complete details of each file is listed. We sincerely hope that you find our responses and modifications satisfactory and consider the manuscript suitable for publication.

**Response to academic editor:**

[1) **Thank you for stating the following financial disclosure:**

**“Our research was supported by the National Natural Science Foundation of China under grant 82474427 to EZ, Jiangsu Natural Science Foundation of China under grant BE2023791 to EZ, Jiangsu Province Leading Talents Cultivation Project for Traditional Chinese Medicine under grant SLJ0319 to EZ, Jiangsu Province Traditional Chinese Medicine Science and Technology Project under grant ZD202007 to EZ and Postgraduate Research & Practice Innovation Program of Jiangsu Province under grant SJCX_0724 to YY.”**

**Please state what role the funders took in the study. If the funders had no role, please state: "The funders had no role in study design, data collection and analysis, decision to publish, or preparation of the manuscript."**

**If this statement is not correct you must amend it as needed.**

**Please include this amended Role of Funder statement in your cover letter; we will change the online submission form on your behalf.]**

**Response:** Thank you for your reminder. We have confirmed that the funders had no role in study design, data collection and analysis, decision to publish, or preparation of the manuscript. The statement has been added in the manuscript and cover letter.

[2) **While revising your submission, please upload your figure files to the Preflight Analysis and Conversion Engine (PACE) digital diagnostic tool, https://pacev2.apexcovantage.com/. PACE helps ensure that figures meet PLOS requirements. To use PACE, you must first register as a user. Registration is free. Then, login and navigate to the UPLOAD tab, where you will find detailed instructions on how to use the tool. If you encounter any issues or have any questions when using PACE, please email PLOS at figures@plos.org. Please note that Supporting Information files do not need this step.]**

**Response:** Thank you for your helpful guidance. The figures have been uploaded to the Preflight Analysis and Conversion Engine (PACE) digital diagnostic tool. PACE generated figure files have been uploaded with the revised submission.

**Response to the reviewer#1’s comments:**

[1) **The authors may wish to expand on how they dealt with duplicate entries and verified each report related SCZ.]**

**Response:** Thank you for your detailed review of our method. The drug name was standardized through the Medex_UIMA_1.8.3 system. Reports suspecting Sodium zirconium cyclosilicate as the primary drug associated with AEs were extracted. The FDA-recommended criteria were followed to remove duplicate entries[1, 2]. For identical CASEIDs, the latest FDA_DT was selected; for similar CASEID and FDA_DT, the record with the higher PRIMARYID was selected[3].

We have refined this part in the data source and data processing section of the manuscript.

References:

1.	Shu Y, He X, Liu Y, Wu P, Zhang Q. A Real-World Disproportionality Analysis of Olaparib: Data Mining of the Public Version of FDA Adverse Event Reporting System. Clin Epidemiol. 2022;14:789-802. https://10.2147/CLEP.S365513 PMID: 35789689

2.	Wang Y, Zhao B, Yang H, Wan Z. A real-world pharmacovigilance study of FDA adverse event reporting system events for sildenafil. Andrology. 2024;12(4):785-92. https://10.1111/andr.13533 PMID: 37724699

3.	Cui Z, Cheng F, Wang L, Zou F, Pan R, Tian Y, et al. A pharmacovigilance study of etoposide in the FDA adverse event reporting system (FAERS) database, what does the real world say? Front Pharmacol. 2023;14:1259908. https://10.3389/fphar.2023.1259908 PMID: 37954852

**[2) It is stated that 8,067,923 AE reports were collected from the FAERS database during the study period and after rigorous data cleaning, 1,154 SZC-related AEs were identified for analysis. The authors may wish to provide details of the so called data cleaning” process, especially with respect to reports related SZC. ( number of reports related to SCZ excluded, the reasons for exclusion etc.)]**

**Response:** Thank you for reading the manuscript so carefully. Considering the drug approval timeline, the FAERS database's DEMO module initially contained 9,440,084 reports from the third quarter of 2018 to the fourth quarter of 2023. Following FDA-recommended deduplication protocols, we removed 1,372,161 duplicate entries, retaining 8,067,923 standardized DEMO records. Subsequently, we conducted systematic searches in the DRUG dataset using standardized medication nomenclature, establishing cross-database linkages through the primaryid field. This identifier enabled targeted extraction of 1,154 case records containing demographic and administrative information related to adverse events from the DEMO database. These validated primaryid entries were further utilized to retrieve 2,027 specific AE reports from the REAC database. The consolidated dataset was then subjected to integrative analysis and critical evaluation.

The absence of granular clinical metadata precluded comprehensive identification and exclusion of data susceptible to potential bias, constituting a fundamental methodological constraint in FAERS-related investigation. Therefore, well-designed clinical studies are needed in the future to investigate causal associations.

**Response to the reviewer#2’s comments:**

**[Minor Issues References: The way references are cited needs to be revised to be presented in the same standard. Citations from periodicals are sometimes written in full and in capital letters, sometimes in abbreviated form and in lowercase letters.]**

**Response:** Thank you very much for your detailed evaluation of our research. We have reviewed and revised the reference list. We have replaced the journal names in the references by substituting the original full journal titles with standardized abbreviated forms that meet the required guidelines, and have additionally supplemented each reference entry with its corresponding PMID number. We also replaced "doi:" with "https//" to enhance compliance with the journal's formatting guidelines. Now we ensure that it is complete and correct. The changes in references are as follows:

1. **Kim MJ, Valerio C, Knobloch GK. Potassium Disorders: Hypokalemia and Hyperkalemia. AMERICAN FAMILY PHYSICIAN. 2023;107(1):59-70.**

We have replaced "AMERICAN FAMILY PHYSICIAN" with "Am Fam Physician" and have added "PMID: 36689973" to complete the information of this reference.

2. **Nilsson E, Gasparini A, Arnlov J, Xu H, Henriksson KM, Coresh J, et al. Incidence and determinants of hyperkalemia and hypokalemia in a large healthcare system. International Journal of Cardiology. 2017;245:277-84. doi: 10.1016/j.ijcard.2017.07.035.**

We have replaced "International Journal of Cardiology" with "Int J Cardiol" and have added "PMID: 28735756" to complete the information of this reference.

3. **Weiss JN, Qu Z, Shivkumar K. Electrophysiology of Hypokalemia and Hyperkalemia. Circulation-Arrhythmia and Electrophysiology. 2017;10(3). doi: 10.1161/circep.116.004667.**

We have replaced "Circulation-Arrhythmia and Electrophysiology" with "Circ Arrhythm Electrophysiol" and have added "PMID: 28314851" and "e004667" to complete the information of this reference.

4. **Llubani R, Vukadinovic D, Werner C, Marx N, Zewinger S, Bohm M. Hyperkalaemia in Heart Failure-Pathophysiology, Implications and Therapeutic Perspectives. Current heart failure reports. 2018;15(6):390-7. doi: 10.1007/s11897-018-0413-9.**

We have replaced "Current heart failure reports" with "Curr Heart Fail Rep" and have added "PMID: 30421355" to complete the information of this reference.

5. **Hoy SM. Sodium Zirconium Cyclosilicate: A Review in Hyperkalaemia. DRUGS. 2018;78(15):1605-13. doi: 10.1007/s40265-018-0991-6.**

We have replaced "DRUGS" with "Drugs" and have added "PMID: 30306338" to complete the information of this reference.

6. **Spinowitz BS, Fishbane S, Pergola PE, Roger SD, Lerma EV, Butler J, et al. Sodium Zirconium Cyclosilicate among Individuals with Hyperkalemia A 12-Month Phase 3 Study. CLINICAL JOURNAL OF THE AMERICAN SOCIETY OF NEPHROLOGY. 2019;14(6):798-809. doi: 10.2215/CJN.12651018.**

We have replaced "CLINICAL JOURNAL OF THE AMERICAN SOCIETY OF NEPHROLOGY" with "Clin J Am Soc Nephrol" and have added "PMID: 31110051" to complete the information of this reference.

7. **Jiang Y, Zhou L, Shen Y, Zhou Q, Ji Y, Zhu H. Safety assessment of Brexpiprazole: Real-world adverse event analysis from the FAERS database. JOURNAL OF AFFECTIVE DISORDERS. 2024;346:223-9. doi: 10.1016/j.jad.2023.11.025.**

We have replaced "JOURNAL OF AFFECTIVE DISORDERS" with "J Affect Disord" and have added "PMID: 37956832" to complete the information of this reference.

8. **Shu Y, Ding Y, Liu Y, Wu P, He X, Zhang Q. Post-Marketing Safety Concerns With Secukinumab: A Disproportionality Analysis of the FDA Adverse Event Reporting System. FRONTIERS IN PHARMACOLOGY. 2022;13. doi: 10.3389/fphar.2022.862508.**

We have replaced "FRONTIERS IN PHARMACOLOGY" with "Front Pharmacol" and have added "PMID: 35754494" and "2022;13:862508"to complete the information of this reference.

9. **Wang F, Zhou B, Sun H, Wu X. Proarrhythmia associated with antiarrhythmic drugs: a comprehensive disproportionality analysis of the FDA adverse event reporting system. FRONTIERS IN PHARMACOLOGY. 2023;14. doi: 10.3389/fphar.2023.1170039.**

We have replaced "FRONTIERS IN PHARMACOLOGY" with "Front Pharmacol" and have added "PMID: 37251345" and "2023;14:1170039" to complete the information of this reference.

10. **Sakaeda T, Tamon A, Kadoyama K, Okuno Y. Data Mining of the Public Version of the FDA Adverse Event Reporting System. International Journal of Medical Sciences. 2013;10(7):796-803. doi: 10.7150/ijms.6048.**

We have replaced "International Journal of Medical Sciences" with "Int J Med Sci" and have added "PMID: 23794943" to complete the information of this reference.

11. **Shu Y, He X, Liu Y, Wu P, Zhang Q. A Real-World Disproportionality Analysis of Olaparib: Data Mining of the Public Version of FDA Adverse Event Reporting System. CLINICAL EPIDEMIOLOGY. 2022;14:789-802. doi: 10.2147/CLEP.S365513.**

We have replaced "CLINICAL EPIDEMIOLOGY" with "Clin Epidemiol" and have added "PMID: 35789689" to complete the information of this reference.

12. **Wang Y, Zhao B, Yang H, Wan Z. A real-world pharmacovigilance study of FDA adverse event reporting system events for sildenafil. ANDROLOGY. 2024;12(4):785-92. doi: 10.1111/andr.13533.**

We have replaced "ANDROLOGY" with "Andrology" and have added "PMID: 37724699" to complete the information of this reference.

13. **Cui Z, Cheng F, Wang L, Zou F, Pan R, Tian Y, et al. A pharmacovigilance study of etoposide in the FDA adverse event reporting system (FAERS) database, what does the real world say? FRONTIERS IN PHARMACOLOGY. 2023;14. doi: 10.3389/fphar.2023.1259908.**

We have replaced "FRONTIERS IN PHARMACOLOGY" with "Front Pharmacol" and have added "PMID:37954852" and "2023;14:1259908" to complete the information of this reference.

14. **Singh J. International conference on harmonization of technical requirements for registration of pharmaceuticals for human use. Journal of pharmacology & pharmacotherapeutics. 2015;6(3):185-7. doi: 10.4103/0976-500x.162004.**

We have replaced "Journal of pharmacology & pharmacotherapeutics" with "J Pharmacol Pharmacother" and have added "PMID: 26312010" to complete the information of this reference.

15. **Brown EG. Methods and pitfalls in searching drug safety databases utilising the Medical Dictionary for Regulatory Activities (MedDRA). Drug Safety. 2003;26(3):145-58. doi: 10.2165/00002018-200326030-00002.**

We have replaced "Drug Safety" with "Drug Saf" and have added "PMID: 12580645" to complete the information of this reference.

16. **Zhou Q, Du Z, Qu K, Shen Y, Jiang Y, Zhu H, et al. Adverse events of epidiolex: A real-world drug safety surveillance study based on the FDA adverse event reporting system (FAERS) database. ASIAN JOURNAL OF PSYCHIATRY. 2023;90. doi: 10.1016/j.ajp.2023.103828.**

We have replaced "ASIAN JOURNAL OF PSYCHIATRY" with "Asian J Psychiatr" and have added "PMID: 37949044" and "2023;90:103828"to complete the information of this reference.

17. **Steinhart AH, Hemphill D, Greenberg GR. Sulfasalazine and mesalazine for the maintenance therapy of Crohn's disease: a meta-analysis. The American journal of gastroenterology. 1994;89(12):2116-24.**

We have replaced "The American journal of gastroenterology" with "Am J Gastroenterol" and have added "PMID: 7977225" to complete the information of this reference.

18. **van Puijenbroek EP, Bate A, Leufkens HGM, Lindquist M, Orre R, Egberts ACG. A comparison of measures of disproportionality for signal detection in spontaneous reporting systems for adverse drug reactions. Pharmacoepidemiology and Drug Safety. 2002;11(1):3-10. doi: 10.1002/pds.668.**

We have replaced "Pharmacoepidemiology and Drug Safety" with "Pharmacoepidemiol Drug Saf" and have added "PMID: 11998548" to complete the information of this reference.

19. **Song Y, Xu Y-l, Lin Y, Zhao B, Sun Q. Fractures due to Aromatase Inhibitor Therapy for Breast Cancer: A Real-World Analysis of FAERS Data in the Past 15 Years. Oncology Research and Treatment. 2020;43(3):96-102. doi: 10.1159/000505376.**

We have replaced "Oncology Research and Treatment" with "Oncol Res Treat" and have added "PMID: 31945768" to complete the information of this reference.

20. **Cornelius VR, Sauzet O, Evans SJW. A Signal Detection Method to Detect Adverse Drug Reactions Using a Parametric Time-to-Event Model in Simulated Cohort Data. Drug Safety. 2012;35(7):599-610.**

We have replaced "Drug Safety" with "Drug Saf" and have added "PMID: 22702641" to complete the information of this reference.

21. **Kinoshita S, Hosomi K, Yokoyama S, Takada M. Time-to-onset analysis of amiodarone-associated thyroid dysfunction. Journal of Clinical Pharmacy and Therapeutics. 2020;45(1):65-71. doi: 10.1111/jcpt.13024.**

We have replaced "Journal of Clinical Pharmacy and Therapeutics" with "J Clin Pharm Ther" and have added "PMID: 31400296" to complete the information of this reference.

22. **Mazhar F, Battini V, Gringeri M, Pozzi M, Mosini G, Marran AMN, et al. The impact of anti-TNFα agents on weight-related changes: new insights from a real-world pharmacovigilance study using the FDA adverse event reporting system (FAERS) database. Expert Opinion on Biological Therapy. 2021;21(9):1281-90. doi: 10.1080/14712598.2021.1948529.**

We have replaced "Expert Opinion on Biological Therapy" with "Expert Opin Biol Ther" and have added "PMID: 34191656" to complete the information of this reference.

23. **Aldahl M, Jensen A-SC, Davidsen L, Eriksen MA, Hansen SM, Nielsen BJ, et al. Associations of serum potassium levels with mortality in chronic heart failure patients. European Heart Journal. 2017;38(38):2890-6. doi: 10.1093/eurheartj/ehx460.**

We have replaced "European Heart Journal" with "Eur Heart J" and have added "PMID: 29019614" to complete the information of this reference.

24. **Desai NR, Kammerer J, Budden J, Olopoenia A, Tysseling A, Gordon A. The Association of Heart Failure and Edema Events between Patients Initiating SZC or Patiromer. Kidney360. 2024;10.34067/kid.0000000586. doi: 10.34067/kid.0000000586.**

We have replaced "2024;10.34067/kid.0000000586" with "2024;5(12):1835-43" to complete the information of this reference.

25. **Thu Kyaw M, Maung ZM. Hypokalemia-Induced Arrhythmia: A Case Series and Literature Review. Cureus. 2022;14(3):e22940-e. doi: 10.7759/cureus.22940.**

We have added "PMID: 35411269" to complete the information of this reference.

26. **Chan YH, Tsai WC, Ko JS, Yin DC, Chang PC, Rubart M, et al. Small-Conductance Calcium-Activated Potassium Current Is Activated During Hypokalemia and Masks Short-Term Cardiac Memory Induced by Ventricular Pacing. Circulation. 2015;132(15):1377-86. doi: 10.1161/circulationaha.114.015125.**

We have added "PMID: 26362634" to complete the information of this reference.

27. **Montford JR, Linas S. How Dangerous Is Hyperkalemia? Journal of the American Society of Nephrology. 2017;28(11):3155-65. doi: 10.1681/asn.2016121344.**

We have replaced "Journal of the American Society of Nephrology" with "J Am Soc Nephrol" and have added "PMID: 28778861" to complete the information of this reference.

28. **Thompson A, Fleischmann KE, Smilowitz NR, de Las Fuentes L, Mukherjee D, Aggarwal NR, et al. 2024 AHA/ACC/ACS/ASNC/HRS/SCA/SCCT/SCMR/SVM Guideline for Perioperative Cardiovascular Management for Noncardiac Surgery: A Report of the American College of Cardiology/American Heart Association Joint Committee on Clinical Practice Guidelines. Circulation. 2024;10.1161/cir.0000000000001285. doi: 10.1161/cir.0000000000001285.**

We have replaced "2024;10.1161/cir.0000000000001285." with "2024;150(19):e351-e442." and have added "PMID: 39316661" to complete the information of this reference.

29. **Raebel MA. Hyperkalemia Associated with Use of Angiotensin-Converting Enzyme Inhibitors and Angiotensin Receptor Blockers. Cardiovascular Therapeutics. 2012;30(3):e156-e66. doi: 10.1111/j.1755-5922.2010.00258.x.**

We have replaced "Cardiovascular Therapeutics" with "Cardiovasc Ther" and have added "PMID: 21883995" to complete the information of this reference.

30. **Imamura T, Oshima A, Narang N, Kinugawa K. Clinical Implications of Sodium Zirconium Cyclosilicate Therapy in Patients with Systolic Heart Failure and Hyperkalemia. JOURNAL OF CLINICAL MEDICINE. 2021;10(23). doi: 10.3390/jcm10235523.**

We have replaced "JOURNAL OF CLINICAL MEDICINE" with "J Clin Med" and have added "PMID: 34884224" and "2021;10(23):5523" to complete the information of this reference.

31. **Paolillo S, Basile C, Aversana SD, Esposito I, Chirico A, Colella A, et al. Novel potassium binders to optimize RAASi therapy in heart failure: A systematic review and meta-analysis. EUROPEAN JOURNAL OF INTERNAL MEDICINE. 2024;119:109-17. doi: 10.1016/j.ejim.2023.08.022.**

We have replaced "EUROPEAN JOURNAL OF INTERNAL MEDICINE" with "Eur J Intern Med" and have added "PMID: 37648583" to complete the information of this reference.

32. **Natale P, Palmer SC, Ruospo M, Saglimbene VM, Strippoli GFM. Potassium binders for chronic hyperkalaemia in people with chronic kidney disease. COCHRANE DATABASE OF SYSTEMATIC REVIEWS. 2020;10.1002/14651858.CD013165.pub2(6). doi: 10.1002/14651858.CD013165.pub2.**

We have replaced "COCHRANE DATABASE OF SYSTEMATIC REVIEWS. 2020;10.1002/14651858.CD013165.pub2(6)." with "Cochrane Database Syst Rev. 2020;6(6):CD013165." and have added "PMID: 32588430" to complete the information of this reference.

33. **Narita Y, Fukumoto K, Fukunaga M, Kondo Y, Ishitsuka Y, Jono H, et al. Comparative Study of Constipation Exacerbation by Potassium Binders Using a Loperamide-Induced Constipation Model. International Journal of Molecular Sciences. 2020;21(7). doi: 10.3390/ijms21072491.**

We have replaced "International Journal of Molecular Sciences. 2020;21(7)." with "Int J Mol Sci. 2020;21(7):2491" and have added "PMID: 32260183" to complete the information of this reference.

34. **Stavros F, Yang A, Leon A, Nuttall M, Rasmussen HS. Characterization of Structure and Function of ZS-9, α K<SUP>+</SUP> Selective Ion Trap. PLOS ONE. 2014;9(12). doi: 10.1371/journal.pone.0114686.**

We have replaced "PLOS ONE" with "PLoS One" , delated "<SUP>" and added "PMID: 25531770" and "e114686" to complete the information of this reference.

35. **Rafique Z, Peacock WF, LoVecchio F, Levy PD. Sodium zirconium cyclosilicate (ZS-9) for the treatment of hyperkalemia. EXPERT OPINION ON PHARMACOTHERAPY. 2015;16(11):1727-34. doi: 10.1517/14656566.2015.1066334.**

We have replaced "EXPERT OPINION ON PHARMACOTHERAPY" with "Expert Opin Pharmacother" and have added "PMID: 26159448" to complete the information of this reference.

36. **Kosiborod M. Effect of Sodium Zirconium Cyclosilicate on Potassium Lowering for 28 Days Among Outpatients With Hyperkalemia: The HARMONIZE Randomized Clinical Trial (vol 312, pg 2223, 2014). JAMA-JOURNAL OF THE AMERICAN MEDICAL ASSOCIATION. 2015;313(5):526-. doi: 10.1001/jama.2014.17494.**

We have replaced this reference with "Kosiborod M, Rasmussen HS, Lavin P, Qunibi WY, Spinowitz B, Packham D, et al. Effect of Sodium Zirconium Cyclosilicate on Potassium Lowering for 28 Days Among Outpatients With Hyperkalemia: The HARMONIZE Randomized Clinical Trial. JAMA. 2014;312(21):2223-33. https://10.1001/jama.2014.15688 PMID: 25402495".

37. **Hida Y, Imamura T, Kinugawa K. Constipation as a Drug-Related Adverse Effect in Patients with Hyperkalemia: Sodium Zirconium Cyclosilicate versus Conventional Potassium Binders. JOURNAL OF CLINICAL MEDICINE. 2023;12(18). doi: 10.3390/jcm12185971.**

We have replaced "JOURNAL OF CLINICAL MEDICINE" with "J Clin Med" and have added "PMID: 37762911" and "2023;12(18):5971" to complete the information of this reference.

38. **Imamura T, Kinugawa K. Successful Conversion from Conventional Potassium Binder to Sodium Zirconium Cyclosilicate in a Patient with Refractory Constipation. MEDICINA-LITHUANIA. 2022;58(5). doi: 10.3390/medicina58050635.**

We have replaced "MEDICINA-LITHUANIA. 2022;58(5)." with " Medicina (Kaunas). 2022;58(5):635." and have added "PMID: 35630052" to complete the information of this reference.

39. **Holleck JL, Han L, Skanderson M, Bastian LA, Gunderson CG, Brandt CA, et al. Risk of Serious Adverse Gastrointestinal Events with Potassium Binders in Hospitalized Patients: A National Study. JOURNAL OF GENERAL INTERNAL MEDICINE. 2024;10.1007/s11606-024-08979-1. doi: 10.1007/s11606-024-08979-1.**

We have replaced "JOURNAL OF GENERAL INTERNAL MEDICINE. 2024;10.1007/s11606-024-08979-1. " with "J Gen Intern Med. Published online August 5, 2024" and have added "PMID: 39103605" to complete the information of this reference.

40. **Driver TH, Shlipak MG, Katz R, Goldenstein L, Sarnak MJ, Hoofnagle AN, et al. Low Serum Bicarbonate and Kidney Function Decline: The Multi-Ethnic Study of Atherosclerosis (MESA). American Journal of Kidney Diseases. 2014;64(4):534-41. doi: 10.1053/j.ajkd.2014.05.008.**

We have replaced "American Journal of Kidney Diseases" with "Am J Kidney Dis" and have added "PMID: 24953891" to complete the information of this reference.

41. **Ash SR, Singh B, Lavin PT, Stavros F, Rasmussen HS. A phase 2 study on the treatment of hyperkalemia in patients with chronic kidney disease suggests that the selective potassium trap, ZS-9, is safe and efficient. KIDNEY INTERNATIONAL. 2015;88(2):404-11. doi: 10.1038/ki.2014.382.**

We have replaced "KIDNEY INTERNATIONAL" with "Kidney Int" and have added "PMID: 25651363" to complete the information of this reference.

42. **Roger SD, Lavin PT, Lerma EV, McCullough PA, Butler J, Spinowitz BS, et al. Long-term safety and efficacy of sodium zirconium cyclosilicate for hyperkalaemia in patients with mild/moderate versus severe/end-stage chronic kidney disease: comparative results from an open-label, Phase 3 study. NEPHROLOGY DIALYSIS TRANSPLANTATION. 2021;36(1):137-50. doi: 10.1093/ndt/gfz285.**

We have replaced "NEPHROLOGY DIALYSIS TRANSPLANTATION" with "Nephrol Dial Transplant." and have added "PMID: 32030422" to complete the information of this reference.

43. **Roger SD, Spinowitz BS, Lerma EV, Singh B, Packham DK, Al-Shurbaji A, et al. Efficacy and Safety of Sodium Zirconium Cyclosilicate for Treatment of Hyperkalemia: An 11-Month Open-Label Extension of HARMONIZE. AMERICAN JOURNAL OF NEPHROLOGY. 2019;50(6):473-80. doi: 10.1159/000504078.**

We have replaced "AMERICAN JOURNAL OF NEPHROLOGY" with "Am J Kidney Nephrol" and have added "PMID: 31658466" to complete the information of this reference.

44. **Roger SD, Spinowitz BS, Lerma EV, Fishbane S, Ash SR, Martins JG, et al. Sodium zirconium cyclosilicate increases serum bicarbonate concentrations among patients with hyperkalaemia: exploratory analyses from three randomized, multi-dose, placebo-controlled trials. NEPHROLOGY DIALYSIS TRANSPLANTATION. 2021;36(5):871-83. doi: 10.1093/ndt/gfaa158.**

We have replaced "NEPHROLOGY DIALYSIS TRANSPLANTATION." with "Nephrol Dial Transplant" and have added "PMID: 32588050" to complete the information of this reference.

45. **Marmol F, Badaruddin M, Baig A, Ye M, Wysocki J, Tahaei E, et al. Fecal ammonium in mice with CKD: gastrointestinal sequestration by sodium zirconium cyclosilicate. AMERICAN JOURNAL OF PHYSIOLOGY-RENAL PHYSIOLOGY. 2023;324(5):F464-F71. doi: 10.1152/ajprenal.00312.2022.**

We have replaced "AMERICAN JOURNAL OF PHYSIOLOGY-RENAL PHYSIOLOGY" with "Am J Physiol Renal Physiol" and have added "PMID: 37022945" to complete the information of this reference.

46. **Fernandez-Prado R, Villalvazo P, Avello A, Gonzalez-de-Rivera M, Aguirre M, Carrasco-Munoz CG, et al. Sodium zirconium cyclosilicate and metabolic acidosis: Potential mechanisms and clinical consequences. BIOMEDICINE & PHARMACOTHERAPY. 2023;158. doi: 10.1016/j.biopha.2022.114197.**

We have replaced "BIOMEDICINE & PHARMACOTHERAPY" with "Biomed Pharmacother" and have added "PMID: 36916426" and "2023;158:114197" to complete the information of this reference.

47. **An JN, Lee JP, Jeon HJ, Kim DH, Oh YK, Kim YS, et al. Severe hyperkalemia requiring hospitalization: predictors of mortality. Critical Care. 2012;16(6). doi: 10.1186/cc11872.**

We have replaced "Critical Care. 2012;16(6)" with "Crit Care. 2012;16(6):R225" and have added "PMID: 23171442" to complete the information of this reference.

48. **Linder KE, Krawczynski MA, Laskey D. Sodium Zirconium Cyclosilicate (ZS-9): A Novel Agent for the Treatment of Hyperkalemia. PHARMACOTHERAPY. 2016;36(8):923-33. doi: 10.1002/phar.1797.**

We have replaced "PHARMACOTHERAPY" with "Pharmacotherapy" and have added "PMID: 27393581" to complete the information of this reference.

49. **Zhang Y, Xu R, Wang F, Liu Y, Xu J, Zhao N, et al. Effects and Safety of a Novel Oral Potassium-Lowering Drug-Sodium Zirconium Cyclosilicate for the Treatment of Hyperkalemia: a Systematic Review and Meta-Analysis. CARDIOVASCULAR DRUGS AND THERAPY. 2021;35(5):1057-66. doi: 10.1007/s10557-020-07134-2.**

We have replaced "CARDIOVASCULAR DRUGS AND THERAPY" with "Cardiovasc Drugs Ther" and have added "PMID: 33459923" to complete the information of this reference.

50. **Packham DK, Rasmussen HS, Lavin PT, El-Shahawy MA, Roger SD, Block G, et al. Sodium Zirconium Cyclosilicate in Hyperkalemia. NEW ENGLAND JOURNAL OF MEDICINE. 2015;372(3):222-31. doi: 10.1056/NEJMoa1411487.**

We have replaced "NEW ENGLAND JOURNAL OF MEDICINE" with "N Engl J Med" and have added "PMID: 25415807" to complete the information of this reference.

51. **Lepage L, Desforges K, Lafrance J-P. New drugs to prevent and treat hyperkalemia. CURRENT OPINION IN NEPHROLOGY AND HYPERTENSION. 2016;25(6):524-8. doi: 10.1097/MNH.0000000000000272.**

We have replaced "CURRENT OPINION IN NEPHROLOGY AND HYPERTENSION" with "Curr Opin Nephrol Hypertens" and have added "PMID: 27584929" to complete the information of this reference.

52. **Wang J, Jin MY, Zheng J, Chen YJ, Sun T, Zheng YF, et al. Sociodemographic and clinical factors influencing serum potassium concentration: A retrospective cohort study. Computational and Structural Biotechnology Journal. 2024;24:1-11. doi: 10.1016/j.csbj.2023.11.044.**

We have replaced "Computational and Structural Biotechnology Journal" with "Comput Struct Biotechnol J" and have added "PMID: 38089009" to complete the information of this reference.

53. **Shukri MZ, Tan JW, Manosroi W, Pojoga LH, Rivera A, Williams JS, et al. Biological Sex Modulates the Adrenal and Blood Pressure Responses to Angiotensin II. Hypertension. 2018;71(6):1083-90. doi: 10.1161/hypertensionaha.117.11087.**

We have added "PMID: 29686001" to complete the information of this reference.

54. **Houghton LA, Heitkemper M, Crowell MD, Emmanuel A, Halpert A, McRoberts JA, et al. Age, Gender, and Women's Health and the Patient. Gastroenterology. 2016;150(6):1332-+. doi: 10.1053/j.gastro.2016.02.017.**

We have added "PMID:27144622" to complete the information of this reference.

55. **Jung H-K, Kim D-Y, Moon I-H. Effects of gender and menstrual cycle on colonic transit time in healthy subjects. The Korean journal of internal medicine. 2003;18(3):181-6. doi: 10.3904/kjim.2003.18.3.181.**

We have replaced "The Korean journal of internal medicine" with "Korean J Intern Med" and have added "PMID: 14619388" to complete the information of this reference.

56. **Verkuijl SJ, Meinds RJ, Trzpis M, Broens PMA. The influence of demographic characteristics on constipation symptoms: a detailed overview. Bmc Gastroenterology. 2020;20(1). doi: 10.1186/s12876-020-01306-y.**

We have replaced "Bmc Gastroenterology" with "BMC Gastroenterol" and have added "PMID: 32493265" and "2020;20(1):168." to complete the information of this reference.

57. **Chiarelli P, Brown W, McElduff P. Constipation in Australian women: prevalence and associated factors. International urogynecology journal and pelvic floor dysfunction. 2000;11(2):71-8. doi: 10.1007/s001920050073.**

We have replaced "International urogynecology journal and pelvic floor dysfunction" with "Int Urogynecol J Pelvic Floor Dysfunct" and have added "PMID: 10805262" to complete the information of this reference.

58. **Kepenekci I, Keskinkilic B, Akinsu F, Cakir P, Elhan AH, Erkek AB, et al. Prevalence of Pelvic Floor Disorders in the Female Population and the Impact of Age, Mode of Delivery, and Parity. Diseases of the Colon & Rectum. 2011;54(1):85-94. doi: 10.1007/DCR.0b013e3181fd2356.**

We have replaced "Diseases of the Colon & Rectum" with "Dis Colon Rectum" and have added "PMID: 21160318" to complete the information of this reference.

59. **Shin GH, Toto EL, Schey R. Pregnancy and Postpartum Bowel Changes: Constipation and Fecal Incontinence. American Journal of Gastroenterology. 2015;110(4):521-30. doi: 10.1038/ajg.2015.76.**

We have replaced "American Journal of Gastroenterology" with "Am J Gastroenterol" and have added "PMID: 25803402" to complete the information of this reference.

60. **Lee KMN, Rushovich T, Gompers A, Boulicault M, Worthington S, Lockhart JW, et al. A Gender Hypothesis of sex disparities in adverse drug events. SOCIAL SCIENCE & MEDICINE. 2023;339. doi: 10.1016/j.socscimed.2023.116385.**

We have replaced "SOCIAL SCIENCE & MEDICINE. 2023;339." with "Soc Sci Med. 2023;339:116385" and have added "PMID: 37952268" to complete the information of this reference.

61. **Leroy F, Dauxois J-Y, Theophile H, Haramburu F, Tubert-Bitter P. Estimating time-to-onset of adverse drug reactions from spontaneous reporting databases. Bmc Medical Research Methodology. 2014;14. doi: 10.1186/1471-2288-14-17.**

We have replaced "Bmc Medical Research Methodology. 2014;14." with "BMC Med Res Methodol. 2014;14:17" and have added "PMID: 24490673" to complete the information of this reference.

**62.	Zhang Q, Ding Y, Shu Y, Chen J. A real-world disproportionality analysis of Rucaparib: Post-marketing Pharmacovigilance Data. BMC CANCER. 2023;23(1). doi: 10.1186/s12885-023-11201-w.**

We have replaced "BMC CANCER. 2023;23(1)." with "BMC Cancer. 2023;23(1):745" and have added "PMID: 37568126" to complete the information of this reference.

Thank you very much for your attention and time. We look forward to your response on our submission. Please do not hesitate to contact us if there are any further questions or comments.

Yours sincerely,

Enchao Zhou

Nanjing University of Chinese Medicine

February 11, 2025
